# Non-canonical WNT-signaling controls differentiation of lymphatics and extension lymphangiogenesis via RAC and JNK signaling

**DOI:** 10.1038/s41598-019-41299-7

**Published:** 2019-03-18

**Authors:** Grit Lutze, Anna Haarmann, Jules A. Demanou Toukam, Kerstin Buttler, Jörg Wilting, Jürgen Becker

**Affiliations:** Department of Anatomy and Cell Biology, University Medical School Göttingen, Göttingen, Germany

## Abstract

Development of lymphatics takes place during embryogenesis, wound healing, inflammation, and cancer. We previously showed that Wnt5a is an essential regulator of lymphatic development in the dermis of mice, however, the mechanisms of action remained unclear. Here, whole-mount immunostaining shows that embryonic day (ED) 18.5 Wnt5a-null mice possess non-functional, cyst-like and often blood-filled lymphatics, in contrast to slender, interconnected lymphatic networks of Wnt5a^+/−^ and wild-type (wt) mice. We then compared lymphatic endothelial cell (LEC) proliferation during ED 12.5, 14.5, 16.5 and 18.5 between Wnt5a^−/−^, Wnt5a^+/−^ and wt-mice. We did not observe any differences, clearly showing that Wnt5a acts independently of proliferation. Transmission electron microscopy revealed multiple defects of LECs in Wnt5a-null mice, such as malformed inter-endothelial junctions, ruffled cell membrane, intra-luminal bulging of nuclei and cytoplasmic processes. Application of WNT5A protein to *ex vivo* cultures of dorsal thoracic dermis from ED 15.5 Wnt5a-null mice induced flow-independent development of slender, elongated lymphatic networks after 2 days, in contrast to controls showing an immature lymphatic plexus. Reversely, the application of the WNT-secretion inhibitor LGK974 on ED 15.5 wt-mouse dermis significantly prevented lymphatic network elongation. Correspondingly, tube formation assays with human dermal LECs *in vitro* revealed increased tube length after WNT5A application. To study the intracellular signaling of WNT5A we used LEC scratch assays. Thereby, inhibition of autocrine WNTs suppressed horizontal migration, whereas application of WNT5A to inhibitor-treated LECs promoted migration. Inhibition of the RHO-GTPase RAC, or the c-Jun N-terminal kinase JNK significantly reduced migration, whereas inhibitors of the protein kinase ROCK did not. WNT5A induced transient phosphorylation of JNK in LECs, which could be inhibited by RAC- and JNK-inhibitors. Our data show that WNT5A induces formation of elongated lymphatic networks through proliferation-independent WNT-signaling via RAC and JNK. Non-canonical WNT-signaling is a major mechanism of extension lymphangiogenesis, and also controls differentiation of lymphatics.

## Introduction

The lymphatic vascular system complements the blood vascular system of most vertebrates and man. In contrast to the widely propagated Starling (1896) equation, there is no permanent re-uptake of filtrated blood plasm into the venous micro-vessels of most organs (except kidney, gut and lymph nodes)^1^. Therefore, all interstitial fluid is transported back to the blood stream via the lymphatics^2–4^. Lymphatics possess several important roles in addition to fluid homeostasis, e.g. in immune surveillance and intestinal chylomicron absorption. They are also involved in pathological mechanisms such as the dissemination of tumor cells. It has also been shown that lymphatic endothelial cells (LECs) can present antigens and modulate immune cell activation and function (reviewed in)^5,6^. In recent decades, our understanding of the cellular and molecular processes that regulate the development and function of the lymphatic vascular system has expanded considerably. It has been shown that lymphatic vessels arise not only from pre-existing veins^7^, but also have a non-venous, mesenchymal origin in avian^8,9^, amphibian^10^ and murine embryos^11–13^.

Numerous aspects of embryonic development, including angiogenesis, are regulated by members of the Wingless-type MMTV integration site (WNT) family^14–16^. WNTs constitute a large group of secreted lipid-modified signaling glycoproteins. They are highly conserved across species and until now 19 different WNT ligands have been characterized in humans and mice. They are involved in various developmental processes, e.g. embryonic patterning, cell growth, migration and differentiation. Mutations of *WNTs* are associated with human diseases, including cancer (reviewed in)^17,18^. The WNT signaling pathway involves several receptors and co-receptors, including Frizzled (FZD) receptors, low-density lipoprotein receptor-related protein (LRP), receptor tyrosine kinase-like orphan receptor (ROR), and the related to receptor tyrosine kinase (RYK) receptor. Thereby, diverse combinations of ligands, receptors and/or co-receptors define the activation of multiple downstream signaling cascades. Commonly, these pathways are divided into two main branches: the ‘canonical’ or β-catenin-dependent and the ‘non-canonical’ or β-catenin-independent pathways^19^. The latter can then be subdivided into the planar cell polarity (PCP) pathway and the Ca^2+^-dependent pathway. However, this classification is only a rough guideline because all WNT pathways are densely-networked as well as tissue- and cell-type-specific^20^.

Among the WNT pathways, the β-catenin-dependent has been characterized best. It involves several WNT ligands (e.g. WNT1, WNT3A, WNT8), FZDs and LRP5/6 receptors, and leads to an inhibition of glycogen synthase kinase 3β (GSK-3β). This results in a loss of β-catenin phosphorylation, prevents its degradation by the proteasome, causes its accumulation in the cytoplasm and translocation into the nucleus. Here, β-catenin binds to T-cell factor (TCF)/lymphoid enhancer-binding factor (LEF) and activates the transcription of WNT target genes. Most commonly, the β-catenin-independent pathways do not activate the TCF/LEF transcription factors^20^. Thereby, the WNT/PCP pathway frequently signals via the RhoA, Rac GTPases and the c-Jun N-terminal kinase (JNK). The WNT/Ca^2+^ pathway involves G-proteins, nuclear factor of activated T-cells, Ca^2+^/calmodulin-dependent protein kinase II, and protein kinase C. It is well established that the non-canonical pathway antagonizes functions of canonical ligands^21^, and that some non-canonical ligands bind to LRP5/6 without inducing phosphorylation, which obviously is the basis for mutually antagonistic effects^22–24^.

WNT5A is a prominent member of the WNT/PCP pathway and has been shown to be an important regulator of lymphangiogenesis. Constitutive homozygous knock-out (ko) of Wnt5a in mice induces significant defects in the morphology and function of dermal lymphatics^25^, while only a minute effect was noted when Wnt5a was overexpressed specifically in myeloid cells^26^. However, the intracellular signaling of WNT5A in LECs remained unexplored. Thus, we sought to elucidate the signaling pathways of WNT5A in LECs in greater detail, and study its functions during lymphatic network maturation and differentiation. Our studies show that WNT5A is an indispensable regulator of dermal lymphatic network morphogenesis and differentiation. These effects are independent of proliferation, and are mediated via RAC and JNK signaling.

## Results

### Wnt5a-null mouse embryos show early signs of edema formation

**Figure 1:**
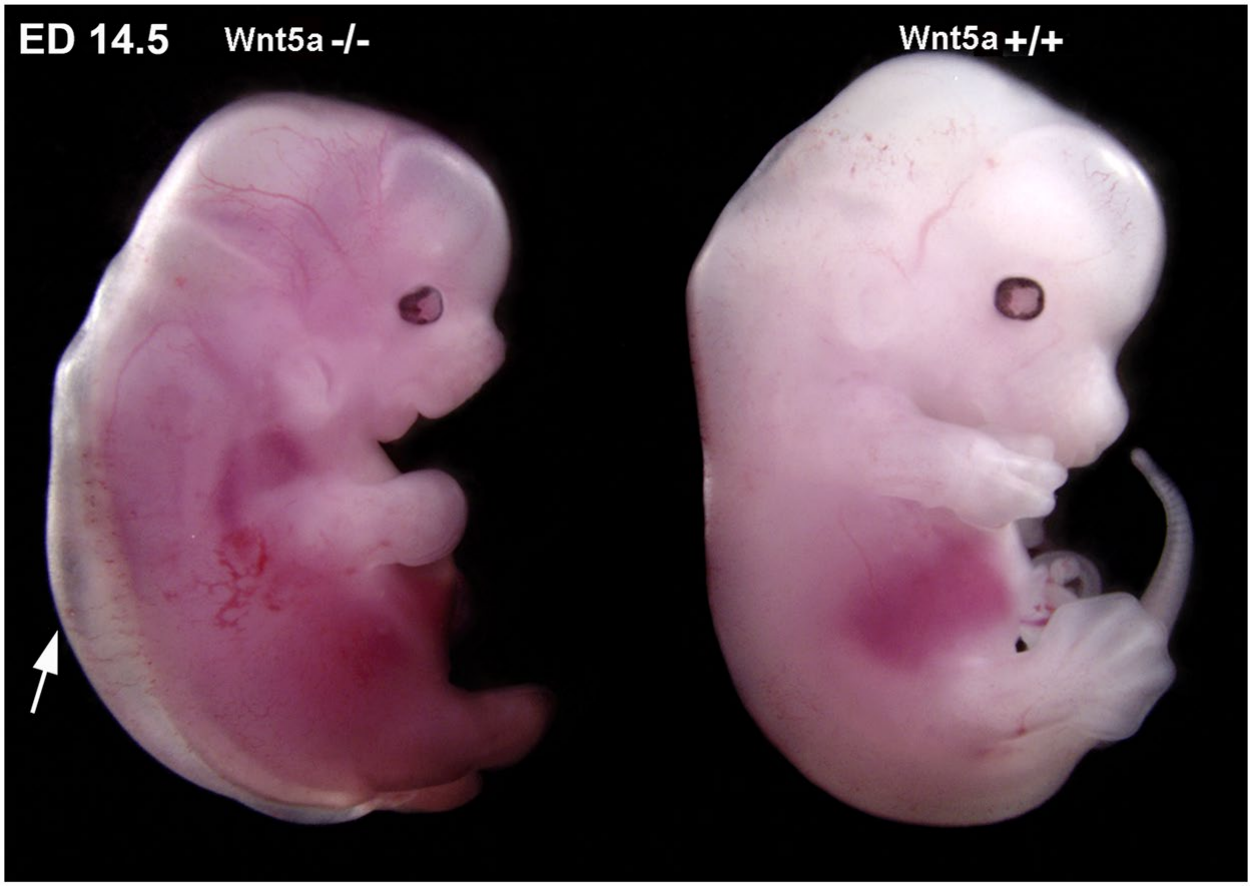
Comparison of ED 14.5 Wnt5a^−/−^ and wt-mice. Note edema formation in the dermis of the back (arrow) of the Wnt5a^−/−^ mouse embryo.

**Figure 2:**
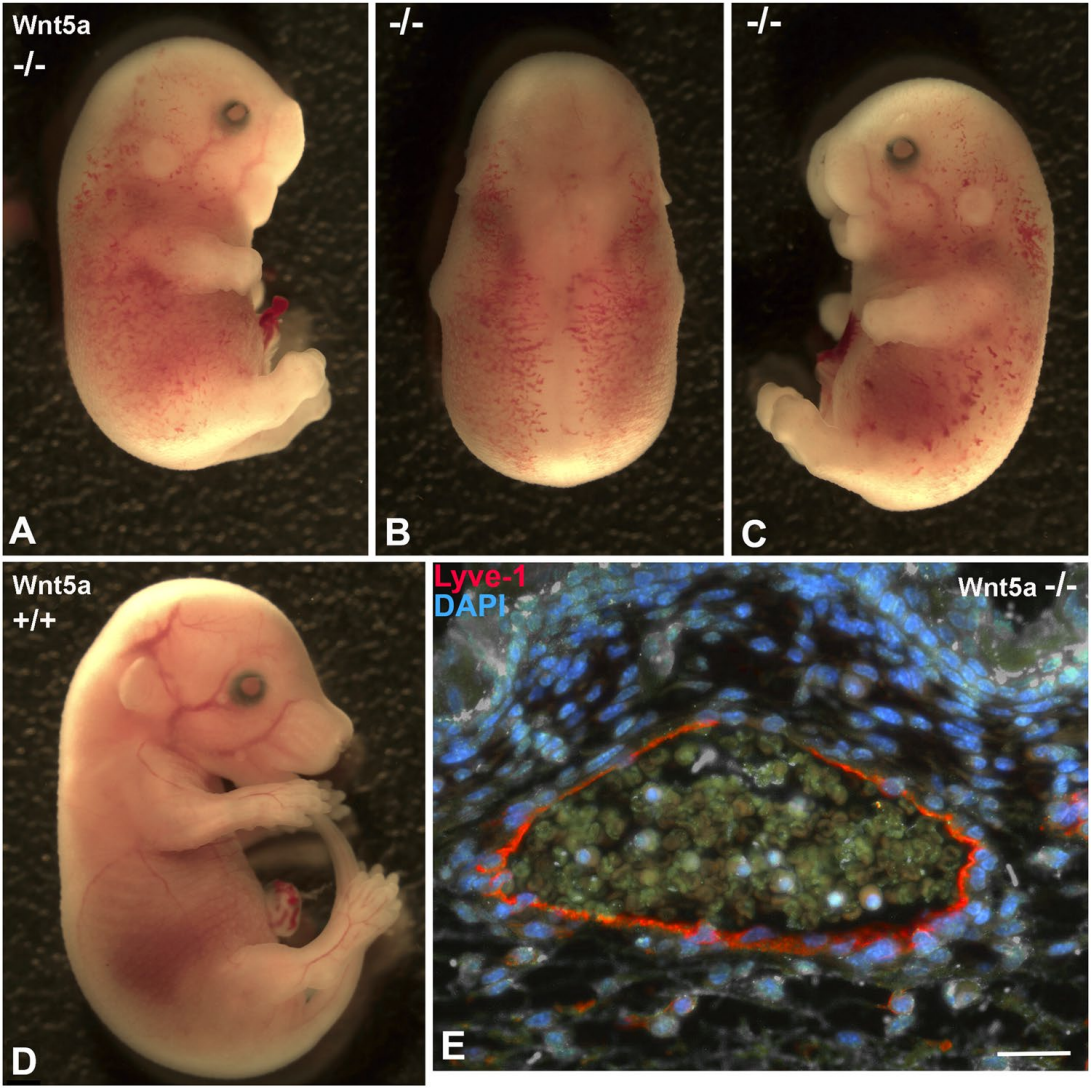
Comparison of ED 17.5 Wnt5a^−/−^ and wt-mice. Note blood-filled lymphatics and petechial dermal bleedings in Wnt5a^−/−^ mice (**A**–**C**) showing different views of one embryo) as compared to wt-embryos of the same litter (**D**). (**E**) Staining with anti-Lyve-1 antibodies (red) reveals that dermal lymphatics are filled with blood cells. Bar = 30 µm.

Most of the multiple defects of Wnt5a-null mouse embryos have been shown quite some time ago^27^. Defects of the dermal lymphatic vascular system were demonstrated exclusively in embryonic day (ED) 18.5 embryos^25^. Since the number of progeny of Wnt5a^+/−^-mice was very low and Wnt5a-null embryos could hardly be recovered especially in late stages of development (there appeared to be a constant loss over time), we regularly crossed Wnt5a^+/−^-mice (BL/6 background) with normal C57BL/6 mice. This recovered the number of progenies significantly. We observed edema formation already in ED 14.5 Wnt5a^−/–^-embryos (Fig. 1). Dermal edema was still present at ED 17.5 and the embryos presented with petechial intra-lymphatic bleedings (Fig. 2A–D), as confirmed by immunostaining with the lymphatic marker Lyve1 (Fig. 2E). Using interstitial injection of 2000kDa FITC-dextran into the paw, Buttler *et al*. showed that lymphatics of ED 18.5 Wnt5a^+/−^ embryos are functional^25^. Here we observed that at ED 16.5 (not shown) and ED 17.5 transport of lymph from the paw is functional in both wild-type (wt)-mice and Wnt5a^+/−^-mice (Suppl. Fig. 1). Thereby, superficial lymphatic collectors were seen to connect the inguinal with the axillary region. In contrast, Wnt5a-null mice never presented a functional lymphovascular system (Suppl. Fig. 1D).

### Wnt5a-null mice possess numerous isolated dermal lymphatic cysts

**Figure 3:**
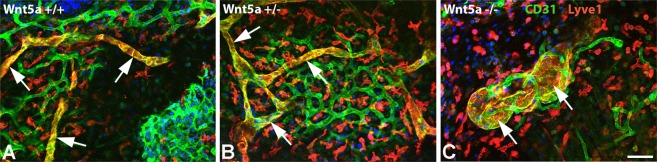
Whole-mount immunostaining of dermal lymphatics of ED 18.5 mouse embryos. Staining with anti-CD31 (green) and anti-Lyve-1 (red) antibodies. Lymphatics (yellow; arrows) are double positive. Note regular, slender lymphatic networks in wt-embryos and Wnt5a^+/−^ -embryos, and cyst-like lymphatics in Wnt5a-null embryos. Blood capillaries (green) are less frequent in Wnt5a-null embryos. Numerous Lyve-1-positive scattered cells are visible (red). Bar = 40 µm.

Previous immunofluorescence studies of cryo-sections revealed dilated lymphatics in ED 18.5 Wnt5a^−/–^-mice, and normal lymphatics in wt- and Wnt5a^+/−^-mice^25^. Here, we studied the lymphatic pattern using whole-mount immunostaining of the dermis. At ED 18.5 we found regular lymphatic networks in wt- and Wnt5a^+/−^-embryos (Fig. 3A,B), and immature sinusoidal or cysts-like lymphatics in Wnt5a-null embryos (Fig. 3C). Obviously, there is a failure of elongation and connectivity of lymphatic anlagen in Wnt5-null embryos. Blood capillaries appeared to be less frequent in these embryos (Fig. 3). Collectively, our data show a massive failure of the development of dermal lymphatic networks in the absence of Wnt5a.

### Proliferation of LECs is unaltered in Wnt5a-null mice

**Figure 4:**
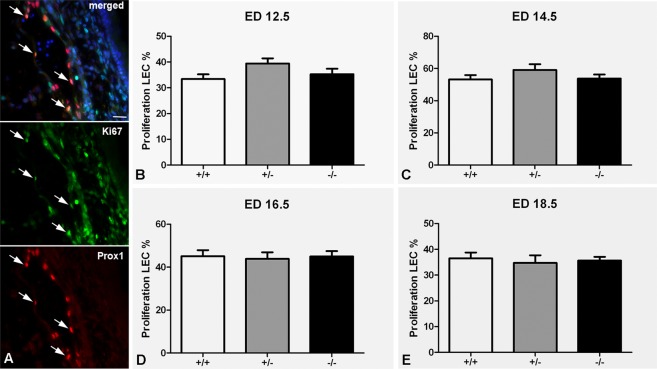
Proliferation studies of LECs in jugular lymph sacs and dermal lymphatics. (**A**) Double immunostaining with anti-Ki67 (green) and anti-Prox1 (red). Representative image showing double-positive LECs, marked with an arrow, in an ED 16.5 dermal lymphatic vessel. Bar = 25 µm. (**B**–**E**) Quantification shows that there are no differences in the proliferation rates between wt-embryos (+/+), Wnt5a^+/−^, and Wnt5a^−/−^ embryos at ED 12.5–ED 18.5. Highest proliferation rates are consistently found at ED 14.5.

In previous studies it was shown that there is no significant difference in proliferation of LECs of ED 18.5 Wnt5a^−/−^ embryos as compared to controls^25^. Here, we studied if earlier stages of development might be affected. We therefore compared proliferation of LECs in jugular lymph sacs and dermal lymphatics with anti-Ki67 and anti-Prox1 double immunostaining in ED 12.5, ED 14.5, ED 16.5, and ED 18.5 embryos of all three genotypes (Fig. 4**)**. There were no differences in the proliferation rates between Wnt5a^−/−^, Wnt5a^+/−^ and wt-embryos in any of the developmental stages. Highest proliferation rates were consistently found at ED 14.5. Our data show that Wnt5a does not regulate LEC proliferation.

### Ultrastructure of Wnt5a^−/−^ lymphatics is abnormal

**Figure 5:**
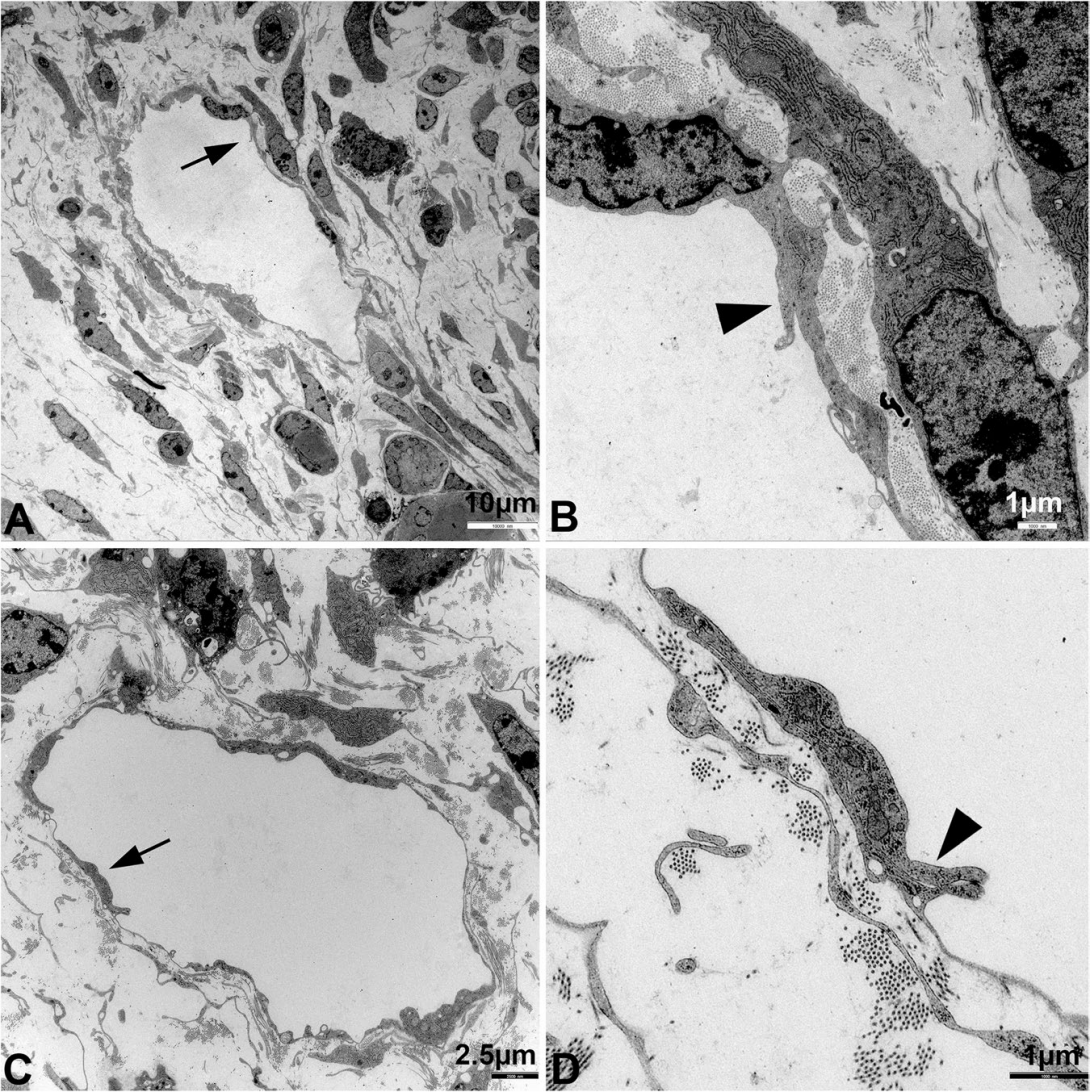
Ultrastructure of initial dermal lymphatics of wt-embryos. Note the typical thin endothelial lining with flat nuclei. Arrows in (**A**,**C**) point to characteristic valve-like junctions shown at higher magnification (arrowheads) in (**B**,**D**), respectively. Note collagen deposits and fibroblasts adjacent to LECs.

**Figure 6:**
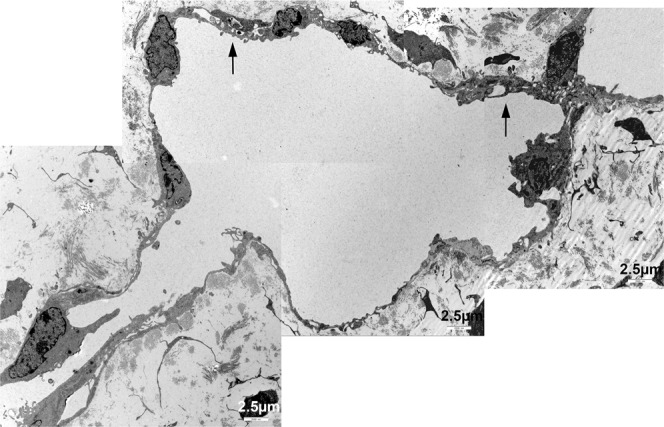
Ultrastructure of initial dermal lymphatics of Wnt5a-null embryos. Note the ruffled endothelial lining, abnormal valve-like junctions with wide inter-endothelial spaces (arrow), and conspicuous bulging of LEC nuclei and cytoplasm into the lumen.

**Figure 7:**
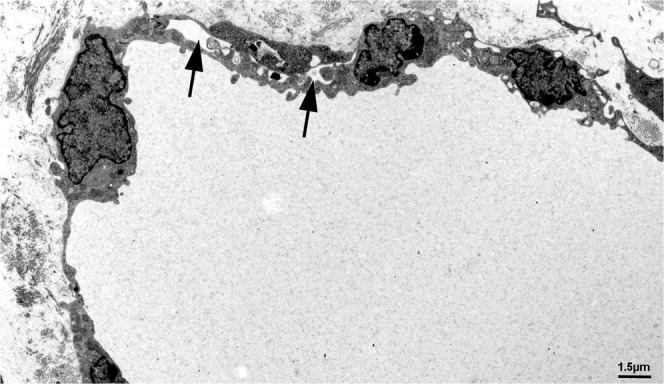
Ultrastructure of initial dermal lymphatics of Wnt5a-null embryos. Higher magnification of Fig. 6 showing ruffled LEC lining and abnormal inter-endothelial spaces (arrows).

Next, we studied the ultrastructure of lymphatics in Wnt5a^−/−^, Wnt5a^+/−^ and wt-embryos at ED 18.5. Thereby, the fine-structure of initial dermal lymphatics of wt-embryos displayed typical characteristics such as thin, flat LECs with overlapping junctions, functioning as delicate valves (Fig. 5). We could not detect any obvious alterations in ED 18.5 Wnt^+/−^ embryos (data not shown). However, in Wnt5a^−/−^ embryos the lymphatics displayed clear alterations. The endothelial lining was ruffled and occasionally discontinuous (Fig. 6**)**. The valve-like, overlapping junctions were not existent. We often observed wide inter-endothelial spaces (Fig. 7**)**. There was conspicuous bulging of LEC nuclei and cytoplasm into the lumen, indicating that flattening and cell-cell communication of LECs was abnormal.

### WNT5A protein rescues lymphatic morphogenesis in Wnt5a^−/−^ mice

**Figure 8:**
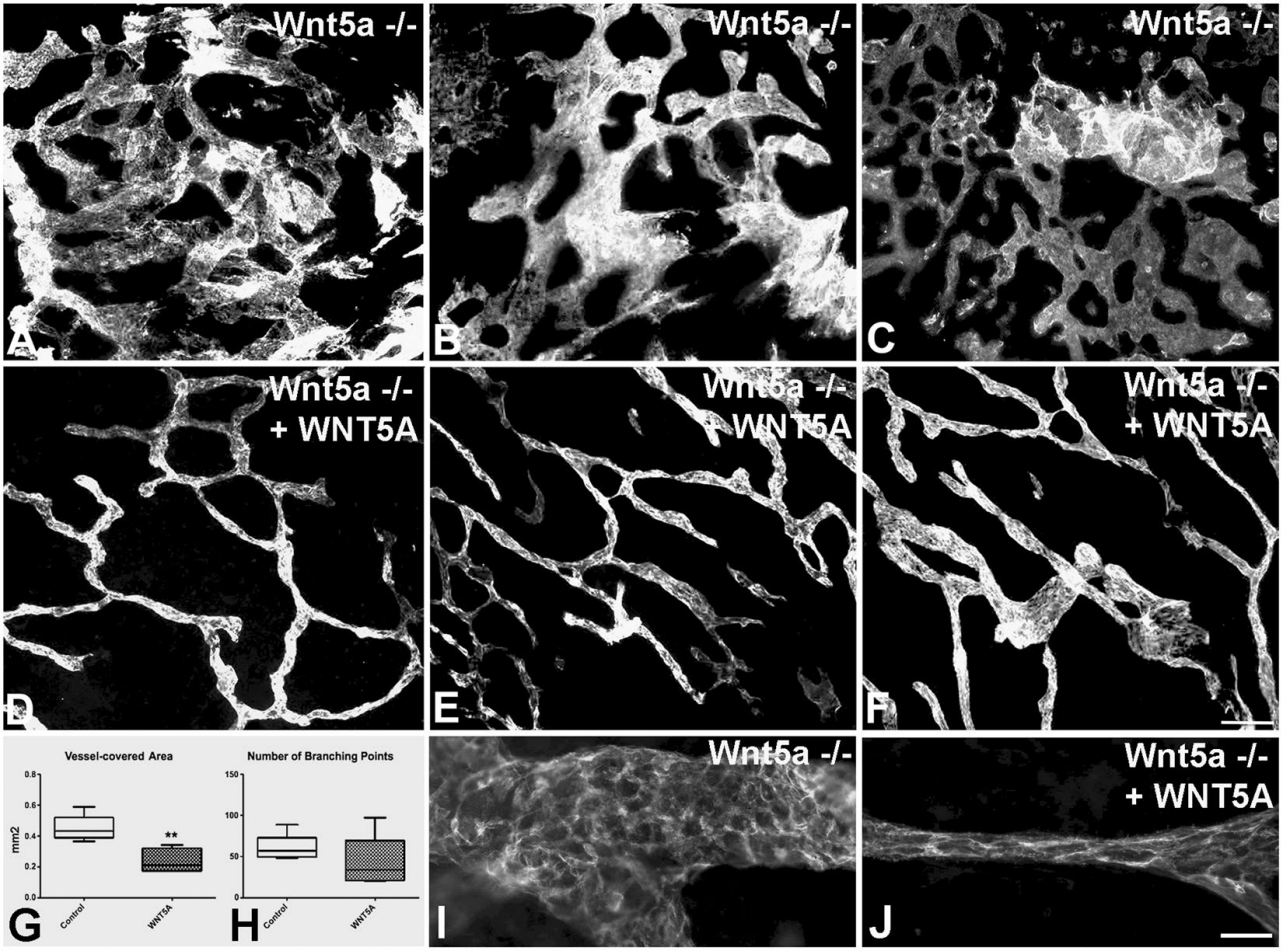
*Ex vivo* treatment of dorsal thoracic dermis from ED 15.5 Wnt5a^−/−^ mice with recombinant WNT5A protein for 2 days. Typical results after staining with anti-Lyve1 antibodies. (**A**–**C**) Control-treated specimens show an immature vascular plexus. (**D**–**F**) WNT5A induced vessel extension and more mature network formation. Bar = 50 µm. (**G**,**H**) Quantification of WNT5A effects reveals significantly decreased vessel-covered area (Mann-Whitney test, p = 0.0079), without statistically significant decrease of the number of branching points (Mann-Whitney test, p = 0.1425). (**I**,**J**) Higher magnifications of (**C**,**F**), respectively, showing wide sweeping morphology of LECs in control-treated specimens (**I**), and longitudinally oriented LECs in WNT5A-treated dermis (**J**). Bar = 15 µm.

Next, we isolated small pieces of dorsal thoracic skin from ED 15.5 Wnt5a^−/−^ mouse embryos and treated the specimens with recombinant human WNT5A protein vs. medium controls. After two days, anti-Lyve-1 whole-mount staining of controls revealed immature lymphatic sinusoids with large vessel diameter and short inter-branching segments (Fig. 8A–C). Application of WNT5A rescued formation of lymphatic networks with slender, elongated vessels; indistinguishable from the lymphatic networks of normal ED17.5 mouse dermis (Fig. 8D–F). Quantification with AngioTool 0.5a software revealed a significant decrease in the vessel-covered area in WNT5A-treated specimens, without a significant change in the number of branching points (Fig. 8G,H). The data show that WNT5A controls the differentiation of sinusoidal lymphatic plexuses into slender, elongated lymphatic networks. Thereby, a change in morphology of LECs became visible. Whereas in the control-treated specimens LECs had a wide sweeping morphology, in the WNT5A-treated dermis they were smaller and oriented longitudinally in the direction of the vessels (Fig. 8I,J).

### Inhibition of Wnt-secretion inhibits lymphatic maturation

**Figure 9:**
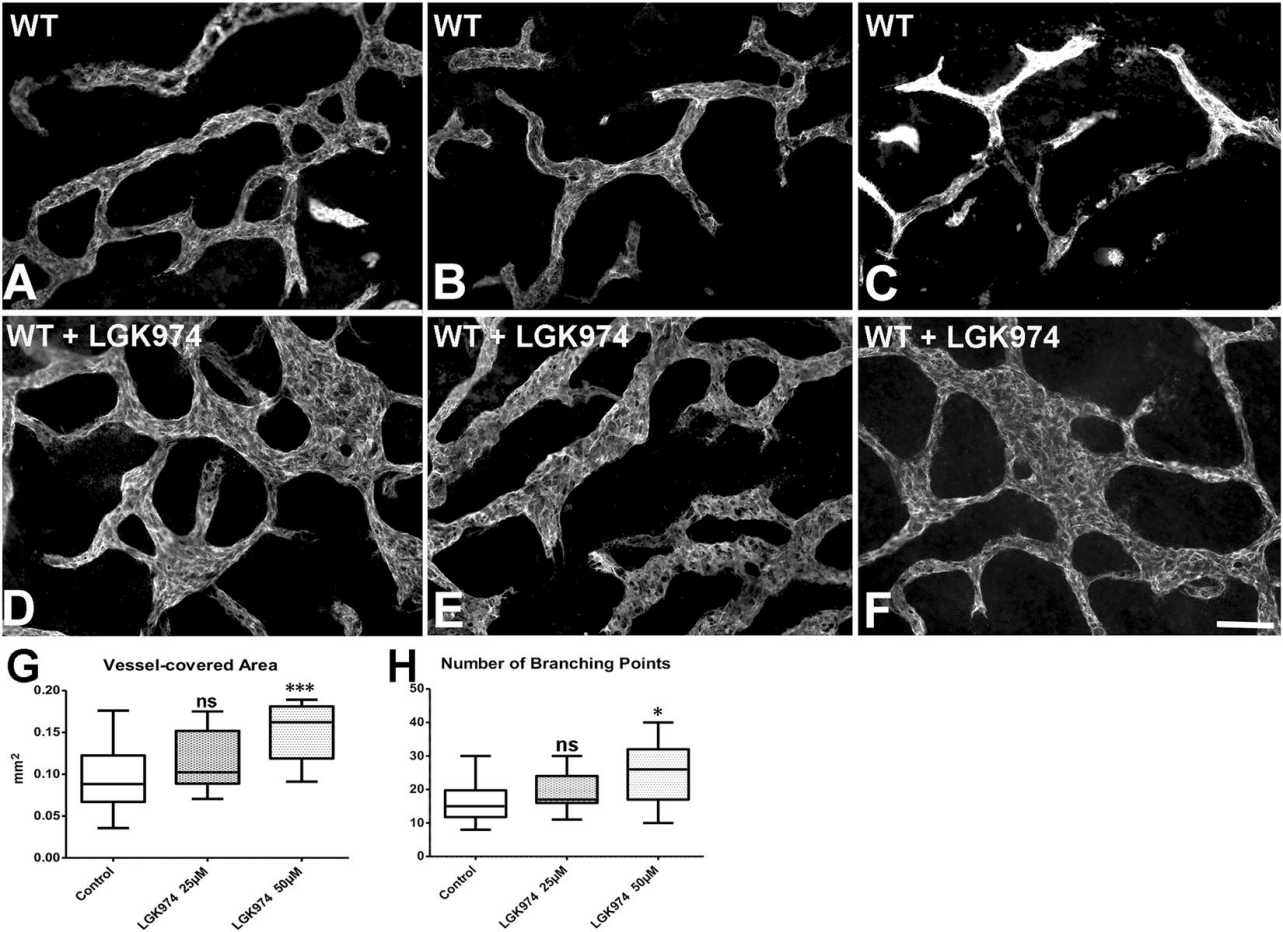
*Ex vivo* treatment of dorsal thoracic dermis from ED 15.5 wild-type-mice with LGK974 for 2 days. Typical results after staining with anti-Lyve1 antibodies. (**A**–**C**) WT-controls; (**D**–**F**) Application of 50 µM LGK974. Bar = 50 µm. Note the increase in vessel-covered area (**G**) and number of branching points (**H**) after 50 µM LGK974, indicating a failure of network maturation. (t-test, LGK974 50 µM p = 0.0004 in (**G**) Mann-Whitney test, LGK974 50 µM p = 0.0244 in (**H**).

In the invers experiment, we treated ED 15.5 wt-mouse dermis with the Wnt-secretion inhibitor LGK974. This compound is a potent inhibitor of porcupine (PORCN), a membrane-bound O-acyltransferase, which palmitoylates WNT ligands. Inhibition of PORCN prevents the export of WNT from the endoplasmic reticulum (ER) and inhibits WNT signaling^28^. LGK974 completely blocked WNT5A secretion in human LECs after 3 days (Suppl. Fig. 2; complete original blot see: Suppl. Fig. 4). Although the effects were milder than those shown in Fig. 8 (most likely due to the storage of Wnts in the extra-cellular matrix), we regularly observed broader, sinusoidal lymphatics in the LGK974-treated specimens (Fig. 9D–F; vs. controls in Fig. 9A–C), indicating a reduction of functionally active Wnt5a during the two-day re-incubation period. Quantification revealed a highly significant increase of the vessel-covered area under 50 µM LGK974, and a mild, significant increase in the number of branching points (Fig. 9G,H). The data provide evidence for the inhibition of lymphatic network maturation after blocking of Wnt-secretion.

### WNT5A induces LEC network formation and tube elongation *in vitro*

**Figure 10:**
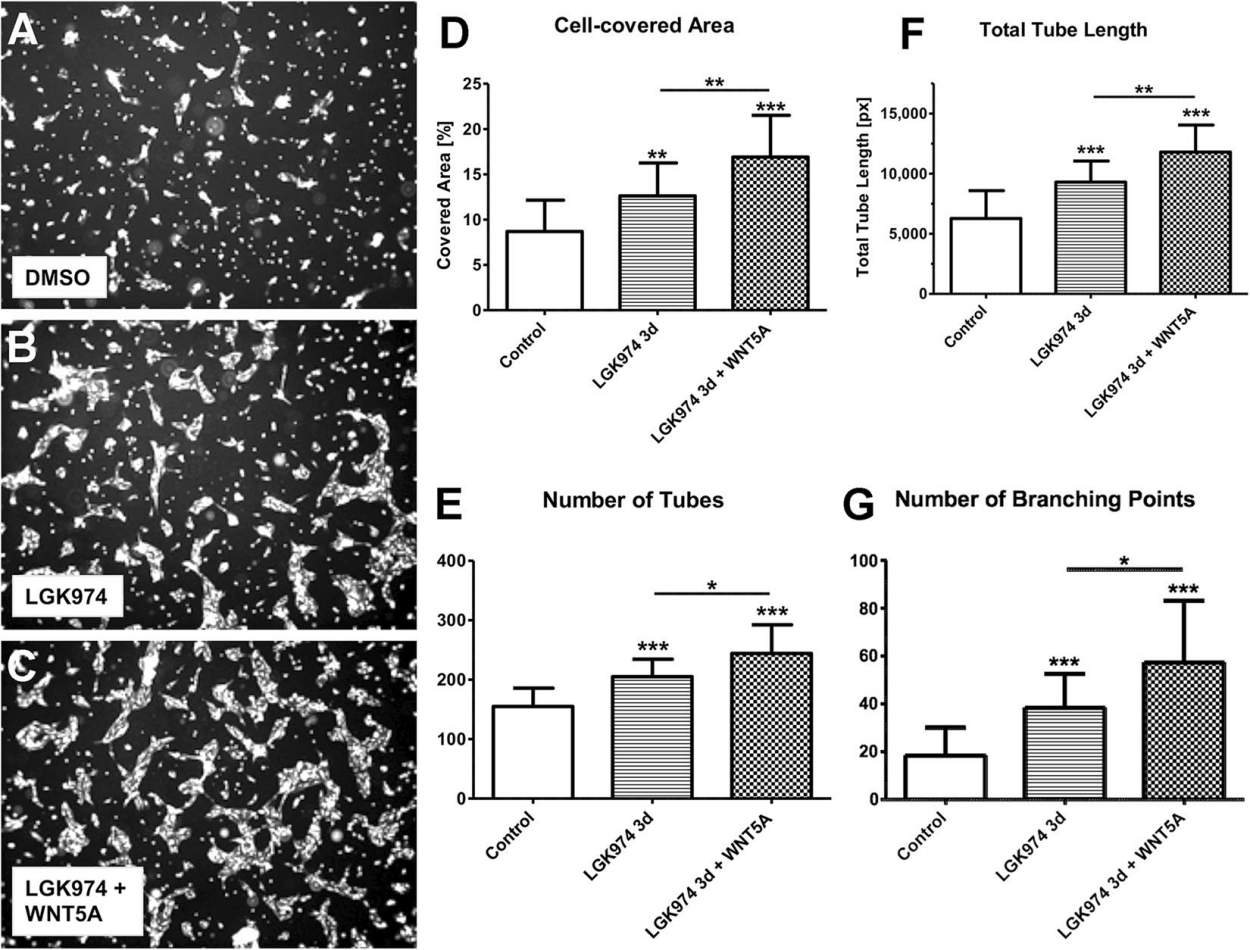
Tube-formation-assay with HD-LECs after pre-treatment with 10 µM LGK974 for 3 days and subsequent application of 500 ng recombinant WNT5A. (**A**–**C**) Representative images of tube-formation-assay with DMSO-controls, HD-LECs pre-treated with LGK974 for 3 d and subsequent application of recombinant WNT5A. Evaluation was performed 9 h after cell seeding. Bar graphs show (**D**) the cell‐covered area, (**E**) number of tubes, (**F**) total tube length, and (**G**) number of branching points. Note the highly significant increase in all aspects of tube formation by WNT5A.

The tube-formation-assays is an established method to measures the ability of scattered endothelial cells seeded on Matrigel to elongate, interconnect and form networks. We studied the behavior of LECs in this assay and compared controls to LECs pre-treated with LGK974 for 3 d, and cells pre-treated with LGK974 and then treated with WNT5A. Data show that LGK974 alone induces network formation **(**Fig. 10A,B**)**, which was an unexpected result, however, WNT5A significantly improved all aspects of network formation **(**Fig. 10C**)**, as revealed by increase of cell-covered area, number of tubes and branching points, and the total length of tubes **(**Fig. 10D–G**)**.

### WNT5A promotes horizontal migration of LECs *in vitro*

The above data have shown that Wnt5a-signaling regulates morphogenesis and differentiation of lymphatics. We then wanted to know, which intracellular signaling pathways are involved. Here, we used the scratch-assay, which can be performed in large numbers to test various activators and inhibitors that affect cell shape and migration of cells in a monolayer.

**Figure 11:**
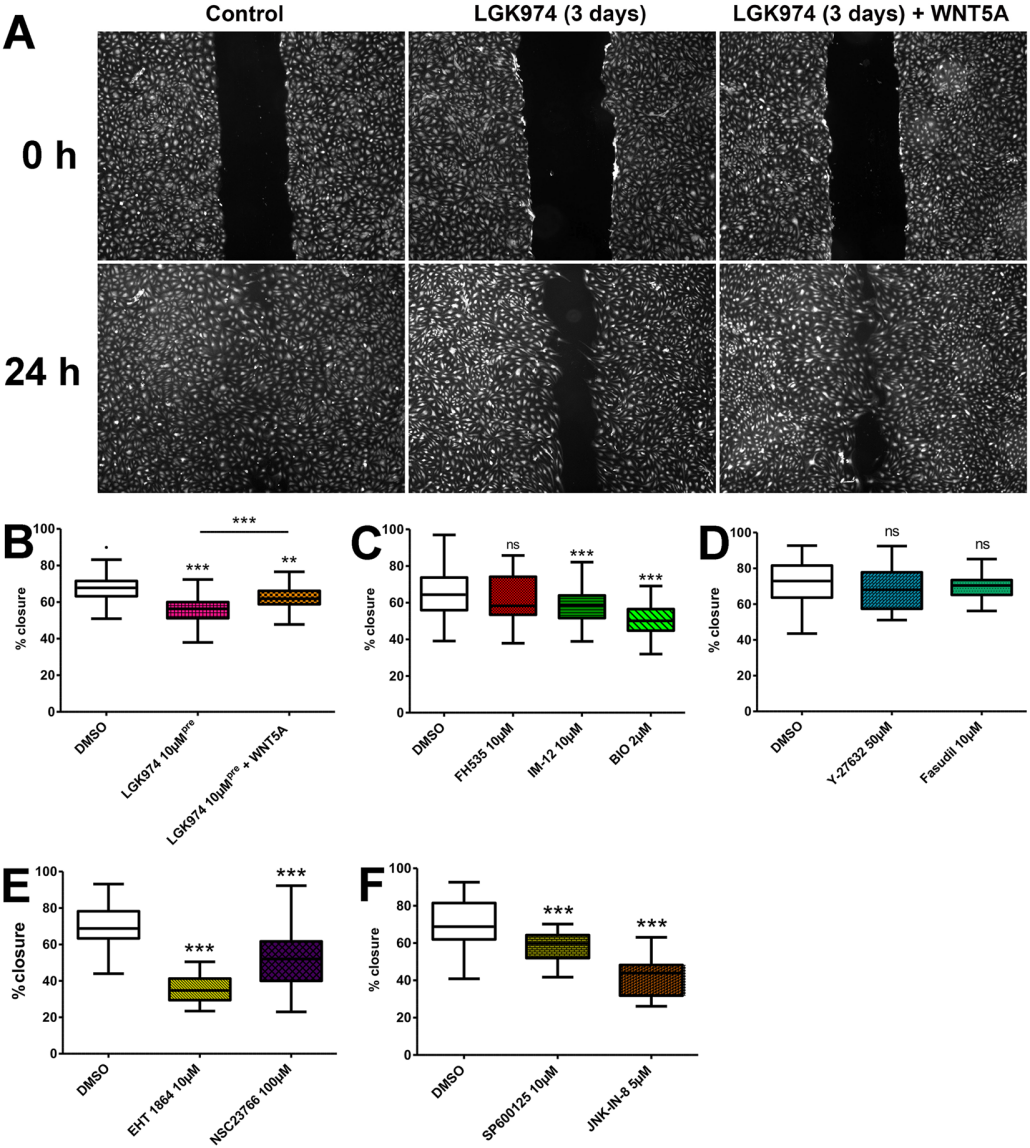
Studies on intracellular WNT-signaling in HD-LECs using small molecules in scratch-assays. (**A**) Scratch assay with HDLECs pre-treated with LGK974 for 3 days and subsequent application of recombinant WNT5A. Representative images after 0 h and 24 h are shown. (**B**) Quantification of scratch closure after 24 hours. Percentage of closure is shown. Inhibition of PORCN with 10 µM LGK974 reduces migration significantly (p < 0.0001). Application of 500 ng recombinant WNT5A increases migration (p = 0.0021). (**C**) Inhibition of the canonical WNT-pathway with FH535 does not affect scratch closure (p = 0.2168). Activation of the canonical WNT-pathway with GSK-3 inhibitors IM-12 or BIO inhibits migration (p = 0.0013 and p < 0.0001, respectively). (**D**) Inhibition of the PCP-pathway by ROCK-inhibitors Y-27632 or Fasudil does not affect scratch closure (p = 0.2904 and p = 0.3595, respectively). (**E**) Inhibition of the PCP-pathway by RAC-inhibitors EHT-1864 or NSC23766 reduces scratch closure (p < 0.0001 and p < 0.0001, respectively). (**F**) Inhibition of the PCP-pathway by JNK-inhibitors SP600125 or JNK-IN-8 reduces scratch closure (p < 0.0001 and p < 0.0001, respectively). At least 3 independent experiments; number of replications see text; t-test: all compared to control, if not stated otherwise.

Firstly, we observed that the WNT-secretion inhibitor LGK974 (10 µM, 25 µM vs. DMSO) significantly inhibited migration and gap closure in a dose-dependent manner (t-test: LGK974 10 µM p = 0.0203 (n = 52), LGK974 25 µM p < 0.0001 (n = 35), all compared to control DMSO (n = 119)). Importantly, LGK974 did not have any effects on proliferation of LECs within 24 h, when the scratch-assays were evaluated (Suppl. Fig. 3A), clearly showing that the assay reliably measures migration. To test whether WNT5A mediates horizontal migration, we used LGK974 pre-incubated HD-LECs and applied recombinant WNT5A to the scratch-assays. Thereby, 500 ng/ml WNT5A significantly improved migration (Fig. 11A,B; t-test: LGK974 10 µM^pre^ p < 0.0001 (n = 25), LGK974 10 µM^pre^ + WNT5A p = 0.0021 (n = 35), all compared to DMSO control (n = 50); LGK974 10 µM^pre^ + WNT5A compared to LGK974 10 µM^pre^ p = 0.0008).

We then tested activators and inhibitors of the canonical WNT-signaling pathway. FH535 is an inhibitor of the β-catenin-mediated pathway by blocking recruitment of β-catenin to β-catenin/TCF-mediated transcription complex. Application of 10 µM FH535 had no effect on migration (Fig. 11C; t-test: FH535 p = 0.2168 (n = 42); DMSO n = 131), although it inhibited proliferation of LECs after 24 h (Suppl. Fig. 3B; two-way ANOVA with Bonferroni post-hoc test, p = 0.0161). This clearly supports the assumption that migration is mediated through PCP-signaling. Interestingly, the two activators of β-catenin-signaling, BIO and IM-12 inhibited HD-LEC migration significantly (Fig. 11C; t-test: IM-12 p = 0.0013 (n = 38), BIO p < 0.0001 (n = 37), DMSO (n = 131)), without having any effects on HD-LEC proliferation after 24 h (Suppl. Fig. 3B**)**. The data show that there is interaction between canonical and non-canonical WNT-signaling.

### Horizontal migration is mediated through RAC and JNK

There are two different arms of down-stream WNT-signaling in the PCP-pathway. One arm involves RHO and rho-associated, coiled-coil-containing protein kinase (ROCK). We applied two commonly used ROCK inhibitors, Y-27632 and Fasudil. Fasudil did not influence proliferation **(**Suppl. Fig. 3C) or migration significantly (Fig. 11D; t-test: Fasudil p = 0.3595 (n = 25); DMSO n = 62)). Application of Y-27632 had no effect on migration (Fig. 11D**;** t-test: Y-27632 p = 0.2904 (n = 26); DMSO n = 62) although it reduced proliferation significantly (two-way ANOVA with Bonferroni post-hoc test, p = 0.0069) (Suppl. Fig. 3C**)**.

The second arm of the PCP-pathway involves Rac family small GTPase (RAC) and JNK. To gain deeper insight into this pathway, we used 4 different drugs to inhibit either RAC (NSC23766 and EHT-1864) or JNK (SP600125 and JNK-In-8). All drugs inhibited migration of HD-LECs very efficiently (Fig. 11E,F; t-test RAC: EHT-1864 p < 0.0001 (n = 34), NSC23766 p < 0.0001 (n = 39), DMSO (n = 67); t-test JNK: SP600125 p < 0.0001 (n = 35), JNK-IN-8 p < 0.0001 (n = 26); DMSO (n = 62)), and only one of them affected proliferation after 24 h (Suppl. Fig. 3D,E; significant decrease after 24 h with NSC23766: two-way ANOVA with Bonferroni post-hoc test, p = 0.0094**)**.

### WNT5A induces transient phosphorylation of JNK

**Figure 12:**
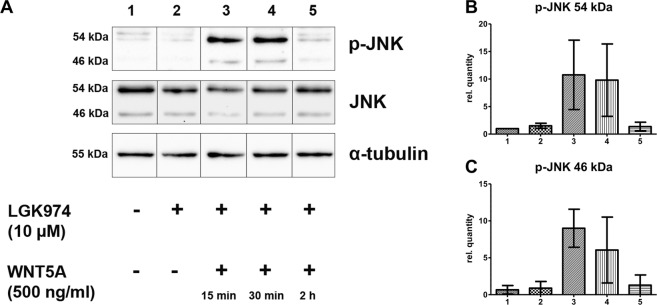
WNT5A induces phosphorylation of JNK. Western blot of HD-LEC lysates. (**A**) Application of WNT5A induces a transient phosphorylation (p) of JNK. (**B**,**C**) Relative quantification of p-JNK at 46 kDa and at 54 kDa. (Mean ± SD from 3 independent experiments.) (Complete original blot see: Suppl. Fig. 5).

**Figure 13:**
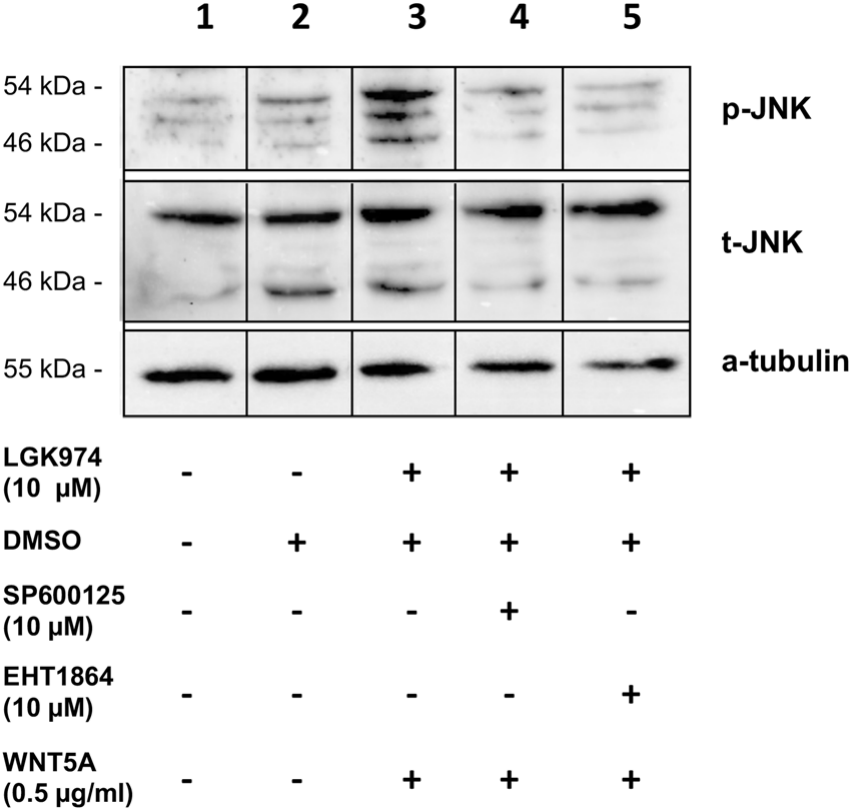
WNT5A-induced JNK phosphorylation is blocked by RAC and JNK inhibitors. Western blot of lysates from HD-LECs treated for 30 min with WNT5A. Lanes 1,2: Negative controls. Lane 3: Induction of p-JNK by WNT5A. Lane 4: Induction of p-JNK is inhibited by the JNK-inhibitor SP600125. Lane 5: Induction of p-JNK is inhibited by the RAC-inhibitor EHT1864. Expression of total JNK (tJNK) and α-tubulin are shown. (Complete original blot see: Suppl. Fig. 6).

Our studies showed that migration of HD-LEC is mediated via RAC and JNK. To examine if WNT5A is capable of activating the JNK pathway, we used LGK974-pre-incubated HD-LECs, stimulated them with recombinant WNT5A and investigated the phosphorylation status of JNK. Since the supplementation of the growth medium with serum led to an overall phosphorylation of JNK, we starved the cells for 24 h in serum-free medium. Our data show that WNT5A induces strong phosphorylation of JNK after 15 min and 30 min, while after 2 h the values decreased to control levels **(**Fig. 12**; c**omplete original blot see: Suppl. Fig. 5). This proves that WNT5A is a potent activator of JNK. We then tested if the induction of JNK phosphorylation can be inhibited by the RAC-inhibitor EHT1864 and the JNK-inhibitor SP600125 (Fig. 13). After 30 min. stimulation with WNT5A, JNK phosphorylation could be detected, which was inhibited by pretreatment with each of the two inhibitors. (Complete original blot see: Suppl. Fig. 6). The data strongly suggest that WNT5A acts via RAC and JNK.

## Discussion

Important functions for Wnt-signaling during angiogenesis have been shown quite some time ago. Abnormal development of blood vessels was noted in mice that are negative for the ligands Wnt2a, Wnt5a, Wnt7a/b, and the receptors Frizzled-4 (Fzd4), and Fzd5^15^. Thereby, endothelium-derived Wnts are important regulators of vascular density, endothelial cell (EC) survival and proliferation, as shown in mice with EC-specific deletion of the pan-Wnt-secretion factor Wls/Evi^29^. Here, we observed hypoplasia of blood capillaries in the dermis of ED 18.5 Wnt5a-null mice, which is in line with recent studies showing that Wnt5a promotes vascular network formation in human adipose-derived microvasculature^30^ and induces hypersprouting in mouse retina^31^.

Regarding the lymphatic vascular system, macrophage-derived Wnts have been studied during inflammation-induced lymphangiogenesis in the cornea^32^. Earlier, regulatory functions for the Wnt-planar cell polarity (PCP) protein Cadherin EGF LAG seven-pass G-type receptor 1 (Celsr1) have been observed in the development of valves in lymphatic collectors^33^. The canonical Wnt pathway seems to be involved in lymphatic valve formation, too, as demonstrated by LEC-specific knock-out of β-catenin^34^. However, mutual interactions between canonical and non-canonical Wnt signaling must be considered. In line with previous models^23^, our studies show that canonical Wnt-signaling inhibits the non-canonical pathway. In LECs, the activators of β-catenin-signaling, BIO and IM-12, inhibit LEC migration via the PCP pathway significantly, showing that proliferation and morphogenesis can be regulated by intracellular interactions of the WNT pathways. Studies by Buttler and coworkers^25^ already indicated a morphogenesis defect of lymphatic networks in the dermis of ED 18.5 Wnt5a-null mice, whereas lymphatics of inner organs appeared to be normal. Similarly, abnormal dermal lymphatics were seen in mice after myeloid-cell-specific knock-out (ko) of the pan-Wnt-secretion factor Wls/Evi^26^. However, these abnormalities are very mild. This may be due to the fact that myeloid-cells (including dermal macrophages) represent just one out of numerous cell types in the dermis expressing Wnts. Wnt5a is highly expressed in epidermis and dermis of murine embryos (see: http://www.genepaint.org/cgi-bin/mgrqcgi94), suggesting concerted activity of both paracrine and autocrine Wnt5a signaling. Since it is hardly possible to knock out Wnt5a in each of the cell types specifically, we chose to study dermis of global Wnt5a-knock-out-mice. Previous data have shown that embryonic lymphangiogenesis is controlled by Wnt-signaling, but detailed studies on the activities of Wnt-ligands, receptors and co-receptors in LECs are missing.

### Wnt5a does not affect proliferation of LECs

Data regarding Wnt5a and endothelial proliferation are inconsistent. A weak but statistically not significant proliferative effect of WNT5A has been reported for HUVECs (human umbilical vein endothelial cells)^35^, while the same group observed an inhibitory effect on bovine aortic endothelial cells^36^. In previous studies we observed a small, though statistically not significant, reduction of LEC numbers in the dermis of ED 18.5 Wnt5a-null mice as compared to controls consisting of a pool of both wt- and heterozygous Wnt5a-ko-mice^25^. Here, we followed up on this topic and compared mice of all three genotypes separately from ED 12.5 - ED 18.5. Highest LEC proliferation rates are consistently present in ED14.5 mice, but there are no differences in Ki67 labeling of LECs between the three genotypes. In line with this, application of recombinant WNT5A protein to human LECs, which were treated for 3d with the PORCN-inhibitor LGK974 to prevent autocrine WNT-signaling, does not affect proliferation. Loss of all Wnt-secretion from myeloid cells significantly increases LEC proliferation in mouse embryos^26^, but the involved components have not been characterized. Regulation of lymphangiogenic growth factors by Wnts in myeloid cells may be a likely hypothesis.

Complex networking of WNTs is also seen in proliferative malignancies in the human. Thereby, significant effects of the canonical pathway (β-catenin, Adenomatous-polyposis-coli, axin) have been well documented e.g. for colon cancer^37^. For WNT5A the data are less clear, however, the majority of studies point to a motility enhancing effect of WNT5A, e.g. by epithelial-mesenchymal transformation^38,39^. Our studies on murine and human LECs strongly support the view that non-canonical Wnt5a signaling regulates aspects of cell shape, motility and differentiation, but not proliferation.

### Wnt5a controls differentiation of lymphatics

We observed that both initial lymphatics and lymphatic collectors are not properly developed in Wnt5a-null mice. Interstitially injected FITC-dextran is not transported centripetally in these mice. In contrast, Wnt5a^+/−^ mice (like wt-mice) take up and transport the marker normally. Thereby, functional lymphatics can be observed in the limbs of ED 16.5 embryos (data not shown), and a very efficient transport is found at ED 17.5. Thereby, lymphatic collectors can be seen accompanying the main blood vessels of the limbs. Additionally, superficial lymphatic collectors are present in the dermis of the trunk and connect the inguinal with the axillary region. They are obviously the reason why metastases of tumors injected into the dermis of the dorsal hip of nude mice are regularly found in axillary lymph nodes. In the human, such collectors do not exist.

Differentiation of initial lymphatics is normal in ED 18.5 Wnt5a^+/−^ mice. Like in wt-mice, the ultrastructural characteristics display typical overlapping junctions, which control the influx of interstitial fluid. In Wnt5a-null mice, these junctions are not present. LECs are ruffled and display protrusions, both luminally and abluminally. The lymphatics are dilated (oedemic) and often contain blood cells. These data strongly suggest that Wnt5a is a crucial regulator of differentiation of lymphatic vessels. Similarly, important functions of Wnt-signaling for the specification of lymphatic vessels have been shown in studies on zebrafish. There, the development of lymphatics from a specific niche in the cardinal vein was demonstrated to be dependent on Wnt5b. Astonishingly, Wnt5b thereby activates the canonical, β-catenin-dependent pathway^40^.

### Extension: an underestimated mechanism in lymphangiogenesis

Our studies show that Wnt5a regulates the morphogenesis of dermal lymphatic vessels without influencing proliferation of LECs. The defects of the dermal lymphatics in Wnt5a-null embryos can be described as: (i) failure to form elongated lymphatics with slender lumen and (ii) failure of lymphatic anlagen to connect into networks, resulting in immaturity of lymphatic plexuses and frequent formation of isolated lymphatic cysts. This shows that non-canonical Wnt-signaling in lymphangiogenesis has very similar functions as during early embryogenesis, e.g. during gastrulation. There, the effects of non-canonical Wnt-signaling have been summarized as ‘convergent extension’^41,42^.

Convergence of angioblasts and extension of the primitive vascular plexus into an organotypic network are important morphogenetic mechanisms in both hemangiogenesis and lymphangiogenesis. It has been shown that hemangioblasts emerge from various extra- and intra-embryonic compartments and assemble (converge) into vascular tubes, which form a dense primitive vascular plexus. The lumen of these vessels is very wide, so they have been assigned as sinusoidal, primary or ‘primitive’. Further development of the vascular plexus takes place by a variety of mechanisms, such as the further integration of angioblasts (convergence), fusion of vessels, sprouting, splitting and intussusception^43–46^. However, elongation of vessels (extension) is a widely observed mechanism, which has often been neglected, or obscured by terminology such as pruning or vessel maturation^44^. Thereby, the term pruning reflects the cutting away of branches from a tree, however, this is not what actually happens during vascular maturation. Instead, vessel extension takes place, which is immediately visible in flat mounts of the retinal vasculature, where the primitive vascular plexus in the periphery of the retina matures into the elongated vascular network in central areas^47^.

Similarly, convergence and extension take place during lymphangiogenesis. Lymphangioblasts, either originating from the venous system or from mesenchymal sources^13,48,49^, assemble (converge) into primary lymphatic networks, which mature (elongate, extend) into lymphatic capillaries and collectors. Thereby, the functional significance of Wnt-signaling has been noted earlier^15,25,40^, but has not yet been clearly assigned to convergent extension mechanisms.

### Wnt5a controls morphogenesis of lymphatics via RAC and JNK

Our studies have shown that Wnt5a is a highly potent morphogen for the lymphatic vascular system. The *ex vivo* application of WNT5A protein to dermis of Wnt5a-null mice rescues the development of slender, elongated lymphatic networks. This obviously takes place without any functional flow in these vessels. Later during development, flow appears to be a morphogenic factor for lymphatic collectors^50^. This may involve flow-induced expression of the transcription factor FOXC2, an essential regulator of lymphatic (and venous) valve formation^51^. In tube formation assays, WNT5A significantly improves network formation by increasing the number and length of tubes. However, it was surprising to see that the complete removal of WNT-signaling in this assay induced network formation of LECs. This obviously shows that, although WNT signaling does have an important function in this process, LECs possess alternative signaling pathways to coordinate homophilic cellular interaction. These pathways might involve the glycogen synthase kinase-3β (GSK3β), a highly versatile kinase, which is not only centrally positioned in the WNT-pathway, but also involved in energy metabolism, neurogenesis, and pattern formation^20,52^.

Our systematic analyses of scratch assays with activators and inhibitors of WNT-signaling provided evidence that inhibition of the RHO-GTPase RAC, or the c-Jun N-terminal kinase JNK significantly reduces migration of LECs, and of note, WNT5A potently induces transient phosphorylation of JNK, which can be inhibited by RAC- and JNK-inhibitors. In contrast, inhibition of the protein kinase ROCK has no effect on LEC migration. Together, these data suggest that WNT5A regulates morphogenesis of lymphatics via WNT-PCP-signaling along the RAC and JNK path.

WNT signaling is an important regulator of the development and differentiation of lymphatics, however, nature seems to have provided LECs (and probably other cell types, too), with an alternative program, which steps in in case of complete absence of WNTs. We have speculated that GSK3β might be involved in this pathway, but this needs to be studied in detail. Then, we observed that both initial lymphatics and lymphatic collectors are maldeveloped in Wnt5a-null mice. It is likely that divergent pathways regulate the differentiation of these vessel types, and again the involvement of WNTs and their interaction with other pathways have to be elucidated. Therefore, the impact of WNTs on lymphangiogenesis and lymphangiogenesis-dependent diseases represents an interesting field for future studies.

## Materials and Methods

All methods were carried out in accordance with relevant guidelines and regulations as listed below. Additionally, all experimental protocols on murine embryos and tissues were approved by the animal welfare officer of the University Medical School Goettingen (UMG).

### Murine embryos and genotyping

We used Wnt5a^[tm1Amc]^ B6 mice (The Jackson Laboratory; Bar Harbor, Maine, US) and C57BL/6 mice. All mice were bred in the Central Animal Facility of the University Medical Center Göttingen, with a 12 h dark/light cycle and water and food ad libitum. All prescriptions of the German Animal Welfare Act (TierSchG) and the German regulations on the welfare of animals used for experiments or for other scientific purposes (TierSchVersV) were kept. PCR genotyping was performed as described^25^.

### Immunohistology

Embryos between embryonic day (ED) 12.5 and 18.5 were fixed in 4% phosphate-buffered paraformaldehyde (PFA). Immunofluorescence staining was performed with the following primary antibodies: rat-anti-mouse CD31 (1:100; BD Pharmingen, Franklin Lakes, US), rabbit-anti-mouse Lyve-1 (1:300; ReliaTech, Wolfenbüttel, DE), rabbit-anti-human Prox1 (1:500; ReliaTech). Appropriate Alexa Fluor® conjugated secondary antibodies were used (1:200; Life Technologies, Eugene, US).

### Proliferation studies in embryos

Wnt5a^−/−^, Wnt5a^+/−^ and wild-type (wt)-embryos of ED 12.5, 14.5, 16.5 and 18.5 were fixed in 4% PFA, embedded in tissue-freeze medium and serially sectioned at 16 µm. Double immunofluorescence staining was performed with the primary antibodies: Ki67 (1:100; DAKO, Hamburg, DE) and Prox1 (1:500; ReliaTech). For each developmental day and genotype, serial sections of at least two embryos were studied. The counts for Prox1-positive LECs of jugular lymph sacs and dermal lymphatics were in the range of 450–900 for each developmental day and genotype.

### Transmission electron microscopy (TEM)

Wnt5a^−/−^, Wnt5a^+/−^ and wt-embryos of ED 18.5 were fixed in Karnovsky’s fixative^53^, embedded in Epon resin and contrasted according to standard procedures^54^. Sections of 70 nm thickness were studied with TEM (LEO 906E; Carl Zeiss Microscopy, Jena, DE).

### *Ex vivo* culture of embryonic mouse dermis

Dorsal thoracic skin of freshly isolated Wnt5a^−/−^ mouse embryos was dissected at ED 15.5, cut into pieces of app. 0.3–0.5 mm and cultured on Millicell^®^ cell culture inserts (0.4 µm, 12 mm; Merck Millipore, Billerica, US) with Endothelial Cell Growth Medium MV 2 (with 1% penicillin-streptomycin; PromoCell, Heidelberg, DE) without (controls) or with 500 ng recombinant Wnt5a protein (R&D Systems, Minneapolis, US) at 37 °C and 5% CO_2_. After 24 h, 50 µl fresh culture medium with 100 ng recombinant Wnt5a was added to the experimental group (n = 5). In the controls, only the medium was supplemented with an equal amount of PBS plus 0.1% BSA (n = 5). After 48 h, the dermis was fixed in 4% PFA for 2 h at room temperature, washed 3 × 15 min and blocked with 1% BSA in PBS overnight. Then, samples were stained with anti-Lyve-1-antibodies (1:200; ReliaTech) for 3 days, followed by overnight washing and overnight staining with goat-anti-rabbit Alexa Fluor 594 (1:200; Life technologies) together with DAPI (1:10 000, Thermo Fisher Scientific, Waltham, US). All steps were done under constant shaking at 4 °C. Images were taken with Axio Imager.Z1 (Carl Zeiss), and ‘vessel-covered area’ as well as ‘number of branching points’ were determined with AngioTool 0.5a^55^.

Correspondingly, dorsal thoracic skin of freshly isolated C57BL/6 wt-mouse embryos was dissected at ED 15.5 and treated with the porcupine inhibitor LGK974 (25–50 µM; Selleck Chemicals, Housten, US) to inhibit Wnt secretion (n = 14). Controls were performed with the solvent DMSO (n = 14). Evaluation was performed as described above.

### Fluorescence microlymphangiography

To study the functionality of the lymphatics we used ED16.5–18.5 mouse embryos and injected 50–100 nl of 2000 kDa FITC-dextran (Sigma, Taufkirchen, DE; 8 mg/ml PBS) into the paws of control embryos and the most distal part of the legs of the homozygous Wnt-null embryos (n = 18). Embryos were studied with a fluorescence stereomicroscope (Leica Microsystems, Wetzlar, DE). Images were taken at regular intervals up to 15 min after injection.

### Cell culture

We purchased several Human Dermal Lymphatic Endothelial Cells (HD-LEC) from PromoCell (Heidelberg, DE) and cultured them according to the supplier’s instructions. Purity of the cells was controlled by CD31 and Prox1 immunostaining, and the three cell lines, which presented as almost 100% pure, were used^56^. All experiments were performed on these 3 cell-lines separately and the results pooled for statistical analyses.

### Immunocytology

Cells were cultured on 4-chamber-slides (BD Biosciences, Bedford, US), fixed for 2 min in 4% PFA and incubated with the following primary antibodies: mouse-anti-human CD31 (1:50; BD Pharmingen), Prox1 (1:1000; ReliaTech). Appropriate Alexa Fluor®-conjugated secondary antibodies were used (1:200; Life Technologies).

### *In vitro* cell proliferation

*In vitro* proliferation assays were performed as described previously^57^. 5 × 10^3^ cells per well were seeded in 96-well culture plates with 100 µl culture medium. After 12 hours (h), treatment was applied to the cultures and the controls (t = 0) were fixed. Subsequently cells were fixed at 24 h intervals, stained with crystal violet and analyzed photometrically at 570 nm.

### Cell migration/scratch assay

HD-LEC were seeded into 24-well-plates and grown to confluence. For better documentation, cells were labeled with 1.5 µM CellTracker™ Green CMFDA (Thermo Fischer Scientific, Waltham, US) according to the manufacturer’s instructions, before scratching. Scratches were made with a 100 µl pipette tip and fresh medium with test substances or solvents (control) was added. Photos were taken after with Leica DMI6000 B (Leica Microsystems) after 0 and 24 h. The gap closure was measured with GNU Octave, version 4.0.3 (https://www.gnu.org/software/octave/) and plotted in % (100% = complete closure).

### Tube formation assay

Assays were performed on ibidi µ-Slide Angiogenesis slides (Ibidi, Martinsried, DE) and the protocol provided with the slides was optimized for LECs. 10 µl Matrigel (Corning Inc., Corning, US) were used as coating gel-matrix and allowed to polymerize overnight at 37 °C. 5,000 cells were seeded in each well. The cells were pre-treated with LGK974 for 3 d and labeled with CellTracker™ Green CMFDA (1.5 µM; Thermo Fischer Scientific) before the experiment. After 9 h, formation of networks was documented with Leica DMI6000 B (Leica Microsystems). Quantification of networks was performed with WimTube analysis software (Onimagin Technologies SCA, Córdoba, Spain).

### SDS-page/Western blot

Cells were lysed with RIPA lysis buffer (140 mM NaCl, 1 mM EDTA, 10 mM Tris, pH 8, 1% Triton, 0.1% SDS, 0.1% sodium deoxycholate with 1x cOmplete™ protease inhibitor (Roche, Mannheim, DE) and 1 mM sodium orthovanadate). Protein concentrations ware measured with Bradford protein assay. The lysates were denatured (95 °C for 5 min) and 50 μg protein of each lysate was separated by SDS-PAGE. After electrophoresis, proteins were transferred to PDVF membranes (pore size 0.45; Carl Roth, Karlsruhe, DE), blocked with 5% BSA in TBST for 1 h and immunoblotted with rabbit-anti-JNK, anti-phospho-JNK (both 1:1000 in TBST with 5% BSA, Cell Signaling Technology, Beverly, US), and anti-α-tubulin (1:10000 in TBST with 5% BSA, Santa Cruz Biotechnology, Dallas, US). For detection, appropriate HRP-conjugated secondary antibodies (Santa Cruz) were used and imaged with Clarity Western ECL Subtrate (Bio-Rad Laboratories, Hercules, US) using the ChemiDoc Imaging System (Bio-Rad).

### Activators and inhibitors

Recombinant Human/Mouse WNT5A was purchased from R&D Systems (Minneapolis, US), diluted in PBS with 1% BSA. For experiments with recombinant WNT5A, HD-LECs were pre-treated for three days with 10 µM LGK974 (Selleck Chemicals). We used the following inhibitors: BIO (2 µM, Sigma), EHT 1864 (10 µM, Tocris Bioscience, Bristol, GB), Fasudil (10 µM, Selleck Chemicals), FH535 (10 µM, Tocris Bioscience), IM-12 (10 µM, Selleck Chemicals), JNK-IN-8 (5 µM, Merck Millipore), NSC23766 (100 µM, Selleck Chemicals), SP600125 (10 µM, Santa Cruz Biotechnology), Y-27632 (50 µM, Selleck Chemicals). For all inhibitors DMSO (Sigma) was used as solvent.

### Statistical analyses

All statistical analyses were performed with GraphPad Prism version 5.01 (GraphPad Software, La Jolla, US). All data sets were tested for Gaussian distribution with Shapiro-Wilk normality test. When all data sets passed the test, t-test was used, otherwise we used Mann Whitney test (migration, scratch and tube formation assays). Statistical analyses for proliferation studies were performed with Two-way ANOVA and Bonferroni Post-hoc-test. Differences were considered as significant with *p-value ≤ 0.05, **p ≤ 0.01 and ***p ≤ 0.001.

## Supplementary information

Related Manuscript File

## Data Availability

All data are included in the manuscript and the supplemental figures.
