# Supplementary material for: Non-canonical WNT-signaling controls differentiation of lymphatics and extension lymphangiogenesis via RAC and JNK signaling
Source: Sci Rep. 2019 Mar 18;9:4739. doi: 10.1038/s41598-019-41299-7 (PMC6426866; doi:10.1038/s41598-019-41299-7)

**Non-canonical WNT-signaling controls differentiation of lymphatics and extension lymphangiogenesis via RAC and JNK signaling**

Grit Lutze, Anna Haarmann, Jules A. Demanou Toukam, Kerstin Buttler, Jörg Wilting*, Jürgen Becker

Institute of Anatomy and Cell Biology, University Medical School Göttingen, Göttingen, Germany

*Corresponding author: Prof. Dr. J. Wilting, University Medical School Goettingen, Department of Anatomy and Cell Biology, Kreuzbergring 36, D-37075 Goettingen, Germany, Email: joerg.wilting@med.uni-goettingen.de

**Supplemental Figures**

**Suppl. Fig. 1: Interstitial injection of 2000 kDa FITC-dextran into the paw of ED 17.5 mouse embryos. A – C)** Wnt5a^+/-^-embryos show uptake of the marker into dermal lymphatic networks (shown at higher magnification in **B**) and dermal collectors (shown at higher magnification in **C**) interconnecting the inguinal and axillary regions. Note dilated segments (arrowheads) as indications for valve formation in C). **D**) Wnt5a-null embryos do not possess functional lymphatics. Time after injection is indicated.

**
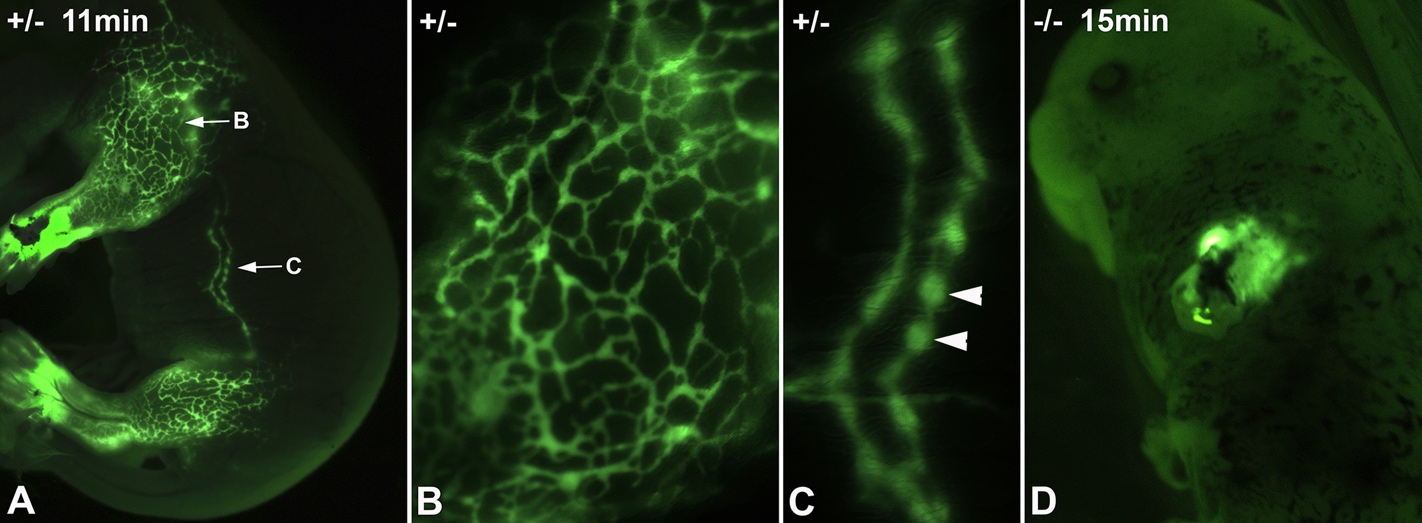
**

**Suppl. Fig. 2: Western blot and Ponceau staining of supernatants from two lines of human dermal-lymphatic endothelial cells (HD-LECc2 and HD-LECc4) after 3-day treatment with LGK974.** Note absence of WNT5A after LGK974 treatment. (Complete original blot see: Suppl. Fig. 4).

**
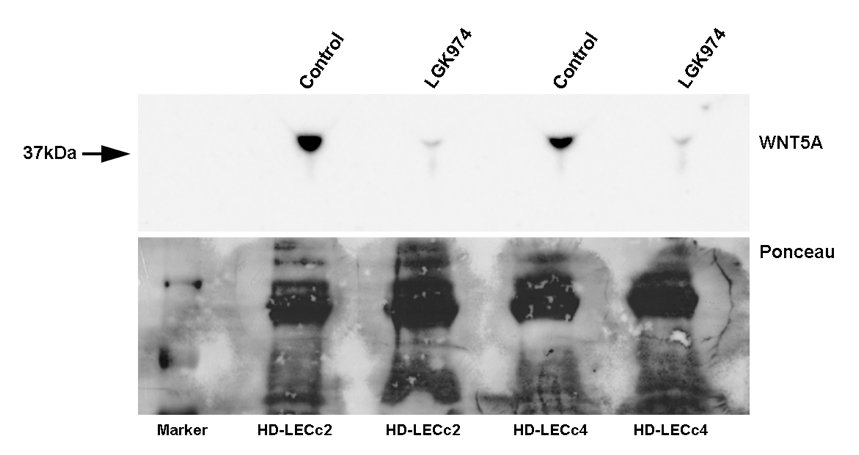
**

**Suppl. Fig. 3: Proliferation studies with HD-LECs treated with activators or inhibitors of the WNT-pathway for 24 h, 48 h and 72 h as compared to DMSO controls.** Treatment with A) LGK974; B) FH535; BIO, IM-12; C) Y-27632, Fasudil; D) EHT 1864, NSC23766; E) SP600125, JNK-IN-8. Note, that stars behind an inhibitor indicates a statistically significant change in the proliferation after 24 hours (when scratch assays are evaluated) compared to the DMSO control (two-way ANOVA with Bonferroni post-hoc test, *p < 0.05, **p < 0.01).

**
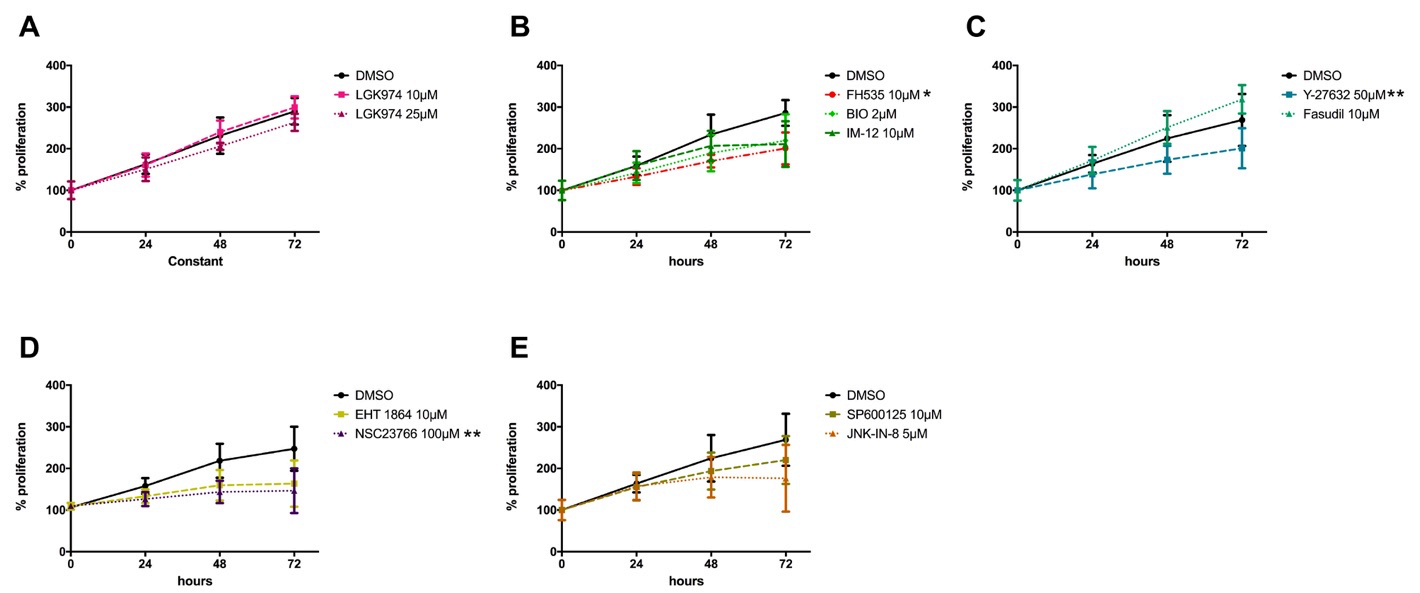
**

**Suppl. Fig. 4: Complete original Western blot and Ponceau staining of supernatants from two lines of human dermal-lymphatic endothelial cells (HD-LECc2 and HD-LECc4) after 3-day treatment with LGK974.** HeLa cell supernatant was used as positive control.


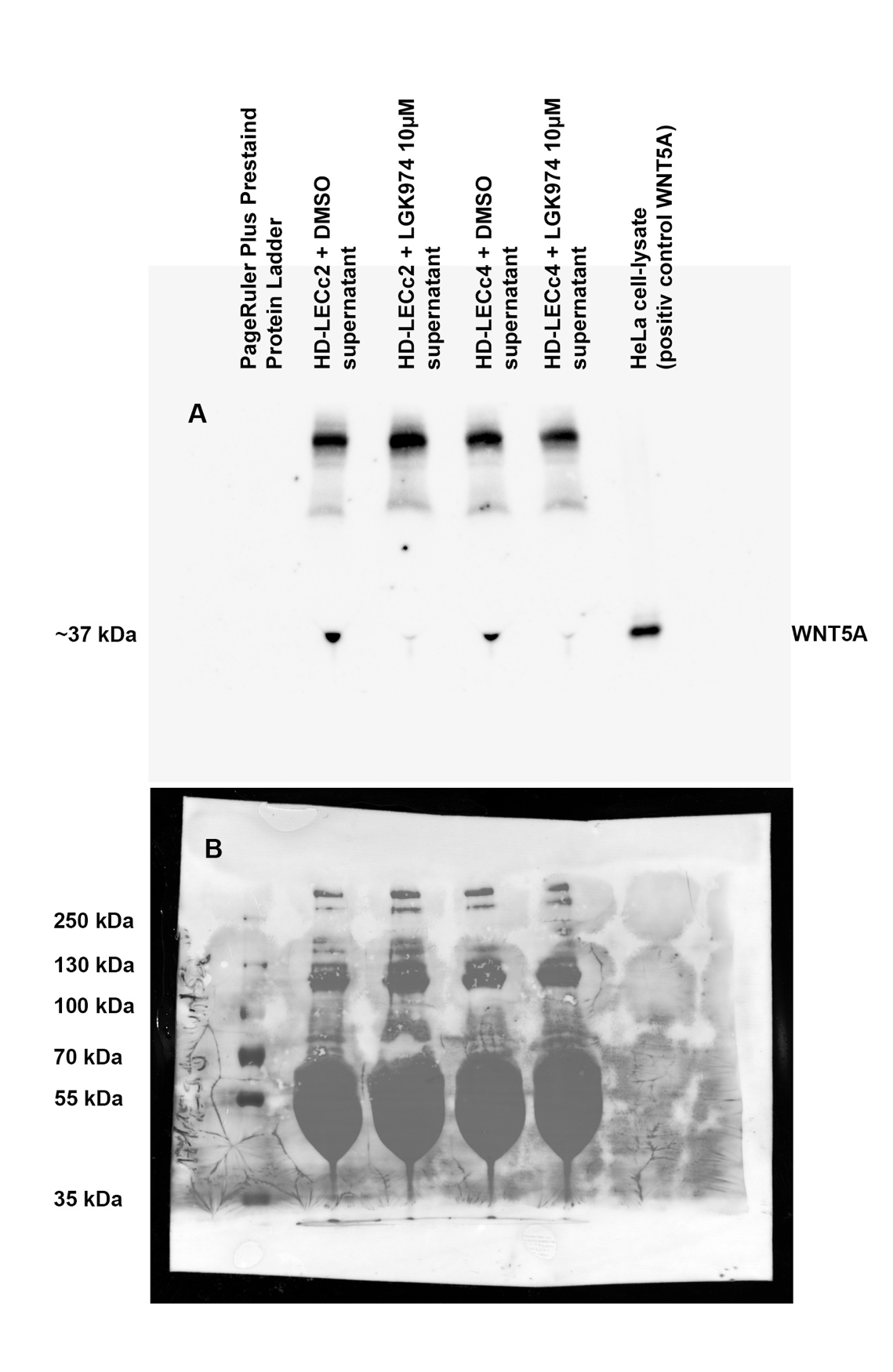


**Suppl. Fig. 5: Complete original Western blots showing staining with antibodies against pJNK, JNK, and α-tubulin.**

**
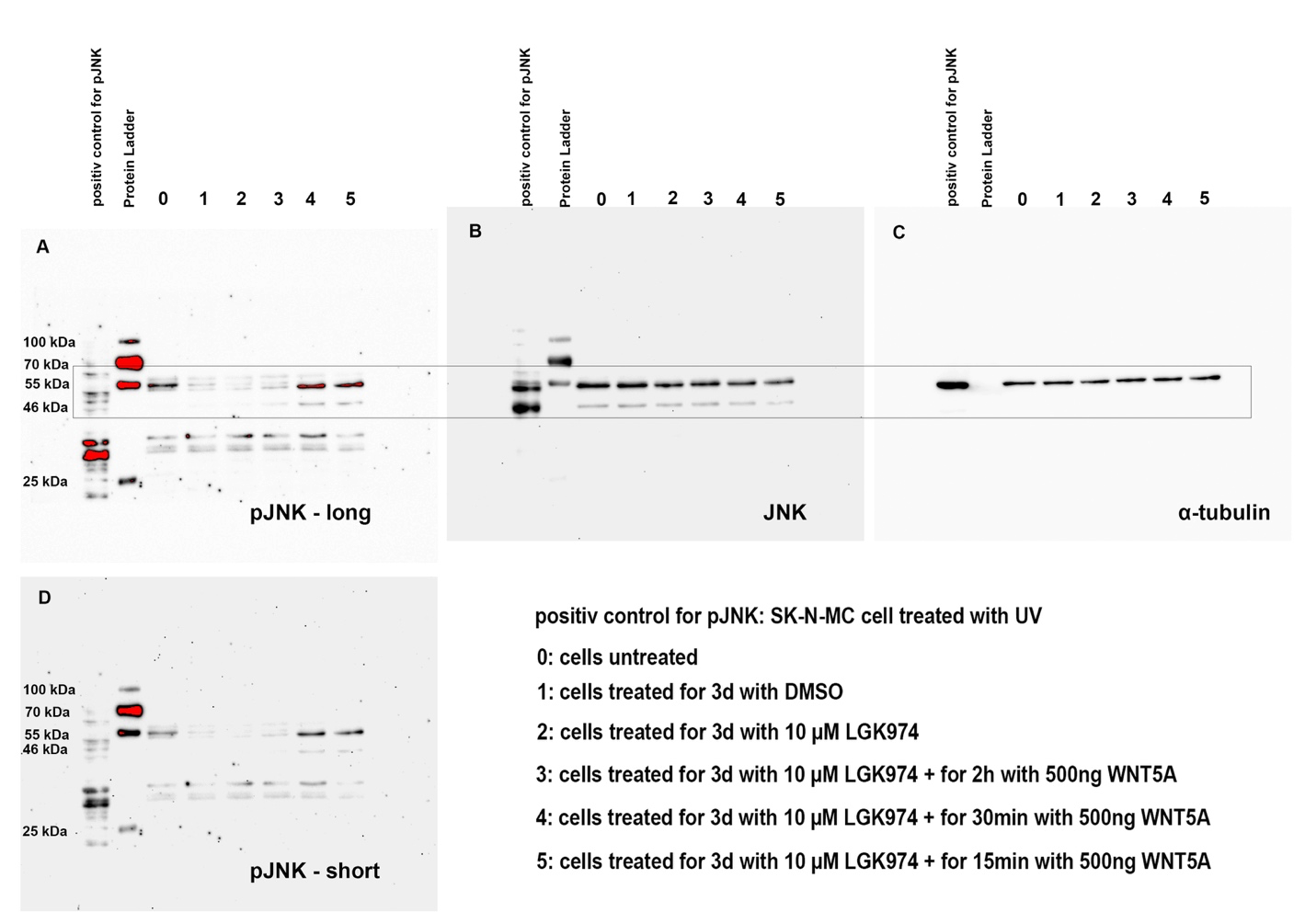
**

**Suppl. Fig. 6: Complete original Western blot showing that WNT5A-induced JNK phosphorylation is blocked by RAC and JNK inhibitors.** Lanes as indicated in Fig. 13.


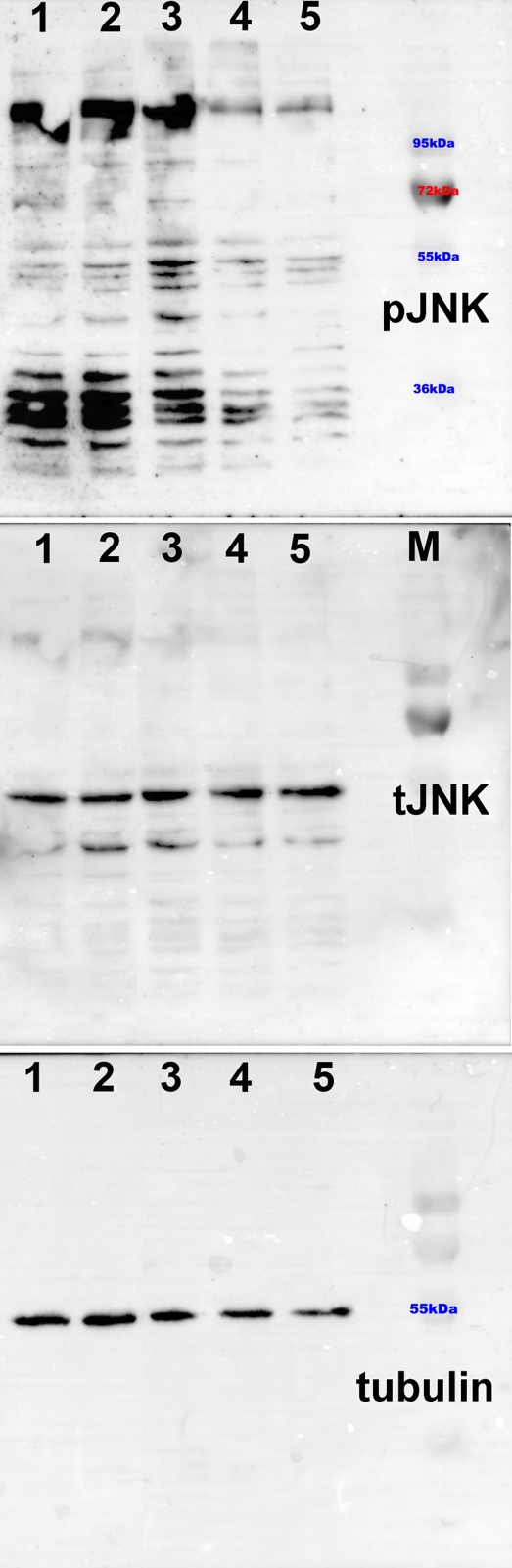

Supplement: Supplementary file 1 — Related Manuscript File [file 41598_2019_41299_MOESM1_ESM.docx]
